# Awareness and acceptance of Jordanian women of egg freezing technology: a cross-sectional study

**DOI:** 10.1080/11287462.2026.2753838

**Published:** 2026-11-03

**Authors:** Dana Al-Zoebi, Omar F. Khabour

**Affiliations:** a Department of Clinical Pharmacy, Faculty of Pharmacy, Jordan University of Science and Technology, Irbid, Jordan; b Department of Medical Laboratory Sciences, Faculty of Applied Medical Sciences, Jordan University of Science and Technology, Irbid, Jordan

**Keywords:** Egg freezing, attitude, knowledge, ethics, Jordan, women

## Abstract

Age-related fertility decline is an escalating challenge for women, especially those balancing career advancement, higher education, or the search for a suitable partner. Despite the availability of preventive options such as elective egg freezing, public awareness and socio-cultural acceptance of this technology remain poorly understood, particularly in developing regions. This study explores Jordanian women’s awareness and perception of elective egg freezing and its associated factors. Using a cross-sectional design, the study targeted adult Jordanian women of reproductive age (*n* = 444) through a convenience sampling approach via social media platforms. Most participants were aware of egg-freezing (91.7%), and 42.8% were positive about it, while only 11.5% were opposed to it. Most respondents justify this technique when chronic diseases, cancer treatments, or surgeries impact a woman's reproductive options. Lower agreement (<50%) was reported in situations such as delaying childbearing due to professional factors and reaching age 35. Age, higher education, high income, marital status, and having children were found to be associated with egg freezing acceptance (*P* < 0.01). In conclusion, Jordanian women generally held positive attitudes toward egg freezing, though medical preservation was favored over social preservation. These insights provide a foundation for developing culturally sensitive educational programs and reproductive health policies in Jordan.

## Introduction

Elective egg freezing has become a popular procedure for healthy women looking to preserve their future reproductive potential (Alteri et al., 2019). Egg freezing was initially introduced to women at risk of fertility loss due to gonadotoxic interventions such as chemotherapy, radiotherapy, or ovarian surgery (Roberts et al., 2015). However, in recent years, egg freezing technology has expanded to encompass healthy, fertile women who wish to delay childbearing for personal, financial, psychological, or career-related reasons, a practice now commonly referred to as social egg freezing (Katsani et al., 2024). This shift is consistent with broader demographic trends, with population-based statistics consistently showing an increase in delayed motherhood, largely attributed to increased female participation in the labor force (O'Rourke et al., 2023). While the medical use of egg freezing is widely recognized on both ethical and clinical grounds, its application for non-medical reasons has generated significant ethical discourse (Borovecki et al., 2018; Bracewell-Milnes et al., 2018; Jones et al., 2020). Delaying reproduction presents significant challenges, as oocyte counts decline, primordial follicles deteriorate, and aneuploidy rates accelerate with age, especially after the mid-30s (Esencan et al., 2022). This highlights the medical basis for offering elective egg freezing before the onset of advanced reproductive age. Thus, egg freezing offers many benefits to a good fraction of women, such as preserving reproductive potential without the significant risk of chromosomal aneuploidy associated with advanced maternal age (van de Wiel, 2022). On the other hand, as a form of assisted reproductive technology, egg freezing carries short-term procedural risks, such as ovarian hyperstimulation syndrome (Mintziori et al., 2019). The “Ethics Committee of the American Society for Reproductive Medicine” indicates that egg freezing is permissible, consistent with the principles of autonomy and ethical justice (Daar et al., 2018). However, women who utilize egg freezing should be adequately educated about the potential benefits, risks, and conditions under which the eggs will be stored (Dondorp & De Wert, 2009).

Several studies have investigated attitudes toward egg freezing across various countries. For example, a study conducted in Germany revealed that the majority of participants had a negative view of egg freezing, while about a third expressed a positive view of the technology (Schick et al., 2017). A survey conducted in Belgium, which included 1,049 women, indicated that 31.5% of them were open to considering egg freezing, while 51.8% of them would not consider it (Stoop et al., 2011). However, data from developing countries are limited (Ghazeeri et al., 2023). A Ghanaian study found that nearly half of the participants (48.9%) were interested in elective egg freezing, even though overall awareness remained low (Osaah et al., 2024). Two qualitative studies highlight the structural barriers to egg freezing, revealing that Turkish participants use it to counter systemic constraints and the lack of a partner, while Iranian women face major hurdles with service accessibility and regulatory navigation (Göçmen & Kılıç, 2018; Moaya et al., 2026).

In Jordan, assisted reproductive technologies, including gamete freezing, are widely accepted and encouraged within Islamic traditions to support family formation (Albeitawi et al., 2026). However, these practices are strictly governed by Sharia law, which permits them only within a valid marriage using the couple's own genetic material while completely prohibiting third-party donations and surrogacy (Shestak et al., 2023). Concurrently, Jordan is experiencing a significant demographic shift as the average age of first marriage for women steadily increases (Rasheed & Tashtoush, 2021). This upward shift is heavily driven by rising rates of higher education among women and their growing engagement in the workforce. Despite these shifting socio-economic dynamics, limited research examines how demographic factors and local trends influence reproductive choices, including emerging technologies like egg freezing. Consequently, the current study aimed to investigate Jordanian women's awareness and attitudes toward egg freezing and its associated factors.

## Methods

### Participants

The study targeted a diverse sample of Jordanian women aged 18–45, as this age group is more likely to consider voluntary egg freezing. The participants were recruited from a demographically diverse population encompassing various income groups (from< 500 to >1000 JOD), educational levels (ranging from school-level to postgraduate degrees), and employment statuses. Non-Jordanian women were excluded from the study. This was achieved by a two-step screening process that was embedded into the online survey tool. First, the introductory page explicitly stated that participation was limited to Jordanian citizens. Second, a mandatory validation question regarding nationality was placed at the absolute beginning of the questionnaire. Utilizing survey skip-logic, any respondent who selected “Non-Jordanian” was immediately redirected to a termination page thanking them for their time, effectively preventing them from accessing or completing the rest of the survey.

The sample size was estimated using GPower-3.1 software. The minimum required sample size was 366 participants. A total of 444 women participated in the study.

### Study design and data collection

The study used a cross-sectional design. Data for this study were collected from April 15 to May 31, 2025, using an anonymous, self-administered Google Forms survey. The questionnaire was distributed digitally to women in Jordan through a combination of email outreach and social media mobilization. Recruitment efforts focused on popular networks in Jordan, including Facebook, WhatsApp, and Instagram, to ensure broad visibility. To optimize the sample size and reach a wider demographic, the study also leveraged snowball sampling by prompting initial respondents to circulate the survey link within their own social and professional circles. The survey was developed based on previously validated questionnaires (Ghazeeri et al., 2023; Schick et al., 2017; Stoop et al., 2011). To evaluate the psychometric properties of the questionnaire, content validation and pilot testing were performed. An expert reviewed the final version of the questionnaire to ensure its content relevance and validity. Following this, a pilot test was conducted with 12 participants who also provided qualitative feedback regarding the instrument's clarity and readability. To establish test-retest reliability, the questionnaire was re-administered to the same 12 participants after a one-week interval. The internal consistency analysis demonstrated acceptable reliability, with Cronbach's alpha values across the different domains exceeding 0.72.

### Ethical approval

The study was approved by the Institutional Review Board at Jordan University of Science and Technology (approval number: 2025/180-10). The survey was anonymous, and the participants were provided with electronic informed consent before completing the survey.

### Data analysis

The SPSS statistical software was used for data analysis. Cross-tabulations and regression models were utilized to assess factors associated with egg freezing technology acceptance. A *P*-value < 0.05 was used as a cutoff point for inferring significance. The dataset supporting the conclusions of this article is included within the article. The raw data are available upon request from the corresponding author.

## Results

Participant demographics are summarized in Table 1. The majority of the respondents held a bachelor's degree (60.6%), were aged 25–34, were employed (52.7%), and reported a monthly household income below 500 Jordanian Dinars (JOD). Additionally, most were single (60.8%), had no children (71.2%), and possessed health insurance (63.1%). Regarding awareness, a substantial majority were familiar with egg freezing technology (91.7%), while 42.8% expressed a positive attitude toward it. Among sources used by participants to gain knowledge about egg freezing technology include social media (52.3%), news channels (36.9%), the internet (34.9%), and school/university curricula (16.5%).

**Table 1.** Demographics of the study sample: frequency and percentage distribution.

Variable	Subcategory	N(%)
**Age**	18–24	123(27.7)
	25–34	231(52.0)
	35–45	90(20.3)
**Education**	High education	94(21.2)
	Bachelor’s degree	269(60.6)
	University student	59(13.3)
	School education	22(5.0)
**Job status**	Full-time job	192(43.2)
	Part-time job	42(9.5)
	Unemployed	250(47.3)
**Income**	Less than 500JD	314(70.7)
	501–1000JD	101(22.7)
	More than 1000JD	29(6.5)
**Health Insurance**	Governmental insurance	162(36.5)
	Private insurance	118(26.6)
	No insurance	164(36.9)
**Marital status**	Married	155(34.9)
	Single	270(60.8)
	Others	19(4.3)
**Having children**	Yes	128(28.8)
	No	316(71.2)
**Are you aware of egg freezing technology**	Yes	407(91.7)
	No	37(8.3)
**Accept egg freezing technology**	Yes	203(42.8)
	No	51(11.5)
	Maybe	190(42.8)
**Sources of information about egg freezing technology**	News channels	164(36.9)
	Family/friends	127(28.6)
	School/university	73(16.5)
	Internet	155(34.9)
	Social media	233(52.3)

Table 2 shows a cross-tabulation analysis of demographic factors and egg freezing acceptance among participants. Age, education, income, marital status, and having children were found to be associated with acceptance of egg freezing technology (*P* < 0.01). The 25–34 age group showed the highest acceptance (51.1%) of egg freezing, while the 18–24 age group showed the lowest acceptance rate (33.3%). The participants with higher education showed higher acceptance than university students (53.2% versus 18.6%, respectively). Furthermore, higher rates of technology acceptance were observed among married individuals, parents, and those in higher income groups (Table 2). With respect to job status and health insurance, no association was observed between these factors and acceptance of the technology (*P* > 0.05, Table 2).

**Table 2.** Chi-square analysis of demographic factors and egg freezing technology acceptance.

Item	Subcategory	Yes: N(%)	Maybe: N(%)	No: N(%)	*P*-value
**Age**	18–24	41(33.3)	67(54.5)	15(12.2)	0.002
	25–34	118(51.1)	93(40.3)	20(8.7)	
	35–45	44(48.9)	30(33.3)	16(17.8)	
**Education**	High-education	50(53.2)	34(36.2)	10(10.6)	<0.000
	Bachelor’s degree	132(49.1)	114(42.4)	23(8.6)	
	University student	11(18.6)	36(61.0)	12(20.3)	
	School education	10(45.5)	6(27.3)	6(27.3)	
**Job status**	Full time	101(52.6)	75(39.1)	16(8.3)	0.088
	Part time	19(45.2)	17(40.5)	6(14.3)	
	Unemployed	83(39.5)	98(46.7)	29(13.8)	
**Income**	<500	125(39.8)	145(46.2)	44(14.0)	0.002
	501–1000	61(60.4)	35(34.7)	5(5.0)	
	>1000	17(58.6)	10(34.5)	2(6.9)	
**Health insurance**	Governmental	73(45.1)	72(44.4)	17(10.5)	0.425
	Private	62(52.5)	44(37.3)	12(10.2)	
	No	68(41.5)	74(45.1)	22(13.4)	
**Marital status**	Married	89(57.4)	47(30.3)	19(12.3)	0.002
	Single	108(40.0)	31(11.5)	31(11.5)	
	Others	6(31.6)	12(63.2)	1(5.3)	
**Have children**	Yes	72(56.3)	41(32.0)	15(11.7)	0.01
	No	131(41.5)	149(47.2)	36(11.4)	

A bivariate nominal regression analysis was performed to pin down factors correlated with egg freezing acceptance (Table 3). Only participants who answered “Yes” or “No” to the acceptance question were included in the analysis. The results showed that university students were less likely to accept this technology (odd ratio [95% CI]: 0.213[0.077–0.592], *P* = 0.003). Similarly, participants with low income (<500JD) were less likely to accept this technology (odd ratio [95% CI]: 0.295 [0.102–0.853], *P* = 0.024). No other relationships were detected with the other examined factors (*P* > 0.05).

**Table 3.** Bivariate nominal regression analysis of variables that impact acceptance of egg freezing technology.

Factor	B	S.E.	Wald	df	Sig.	Exp(B) [95% CI)
**Age**			3.452	2	0.178	
18–24	0.849	0.629	1.820	1	0.177	2.337 [0.681–8.020]
25–34	0.880	0.479	3.369	1	0.066	2.410 [0.942–6.164]
**Education**			8.962	3	0.030	
High	−0.276-	0.450	0.377	1	0.539	0.759 [0.314–1.832]
School	−0.698-	0.679	1.057	1	0.304	0.497 [0.131–1.883]
University student	−1.545-	0.521	8.795	1	0.003	0.213 [0.077–0.592]
**Job status**			1.056	2	0.590	
Full-time job	0.475	0.586	0.657	1	0.417	1.608 [0.51–5.073]
Unemployed	0.092	0.592	0.024	1	0.876	1.097 [0.344–3.496]
**Income (JD)**			5.263	2	0.072	
Less than 500JD	−1.221-	0.542	5.074	1	0.024	0.295 [0.102–0.853]
More than 1000JD	−.480-	0.921	0.272	1	0.602	0.619 [0.102–3.763]
**Health insurance**			1.631	2	0.442	
Governmental	0.410	0.472	0.755	1	0.385	1.506 [0.598–3.796]
No insurance	−.112-	0.450	0.062	1	0.804	0.894 [0.370–2.162]
**Marital status**			0.823	2	0.663	
Married	−.982-	1.249	0.618	1	0.432	0.374 [0.32–4.331]
Single	−1.142-	1.260	0.822	1	0.365	0.319 [0.027–3.769]
**Having Children: No**	−.377-	0.632	0.357	1	0.550	0.686 [0.199–2.366]
**Constant**	2.979	1.505	3.918	1	0.048	19.664

The participants were asked about the situations in which egg freezing technology might be justified (Table 4). A high agreement rate (>80%) was recorded for conditions that might affect reproductive choices, such as the presence of a chronic health condition, cancer treatment, surgical manipulations involving the ovaries, and a family history of early menopause. A lower agreement (<50%) was recorded for situations such as delaying childbearing due to occupational factors and reaching age 35 (Table 4).

**Table 4.** Situations where egg freezing technology might be acceptable, according to the study participants – frequency and percentage distribution.

Item	Yes: N(%)	No: N(%)
Chronic illnesses	413(93.0)	31(7.0)
Maintaining reproductive choices before cancer therapy	400(90.1)	44(9.9)
Maintaining reproductive choices before surgical manipulations of the ovaries	423(95.3)	21(4.7)
Family history of early menopause	360(81.1)	84(18.9)
Waiting for a suitable partner	231(52.0)	213(48.0)
Delaying reproduction due to occupational factors	173(39.0)	271(61.0)
Psychological reasons	192(43.2)	252(56.8)
Reaching the age of 35 without children	176(39.6)	268(60.4)
Reaching the age of 40 without children	240(54.1)	204(45.9)

Concerns of participants about egg freezing technology were explored. Key concerns included lack of knowledge about the technology, risks of bleeding/infection/hormonal changes associated with the procedure (77%–87%), financial cost (78.2%), misuse of stored eggs (83.1), and fear of egg mixing (80.9%). Table 5 provides a full list of recorded concerns.

**Table 5.** Concerns of participants regarding egg freezing technology – frequency and percentage distribution.

Item	Yes	No
**Lack of knowledge**	316(71.2)	128(28.8)
**Fear from bleeding/infection**	341(76.8)	103(23.2)
**Risk associated with hormonal treatment**	386(86.9)	58(13.1)
**Fear of loss of virginity**	234(52.7)	210(47.3)
**Financial cost**	347(78.2)	97(21.8)
**Feel of comfortless**	236(53.2)	208(46.8)
**Conflict with future partner**	241(54.3)	203(45.7)
**Fear of eggs mixing**	359(80.9)	85(19.1)
**Misuse of stored eggs**	369(83.1)	75(16.9)
**Lack of trust**	320(72.1)	124(27.9)

## Discussion

This study investigated the awareness, perceptions, and associated factors regarding elective egg freezing among Jordanian women. The results indicate a high level of awareness of this technology. Furthermore, a substantial proportion of participants expressed positive attitudes toward the procedure, with egg freezing for medical reasons finding greater acceptance than for social reasons.

In recent years, egg freezing has become a popular fertility-preserving procedure for women (Alteri et al., 2019). The study results indicated that 91.7% of women were aware of egg freezing. Similarly, high awareness rates were recorded in studies conducted in the United Kingdom/Denmark (83%), Lebanon (65%), and Ireland (60%) (Ghazeeri et al., 2023; Lallemant et al., 2016; O'Brien et al., 2017). However, lower awareness rates were reported in studies conducted in the United States (29.8%), Italy (34.3%), and Ghana (56.3%) (Milman et al., 2017; Osaah et al., 2024; Tozzo et al., 2019). The relatively high awareness observed in the current study can be attributed to the fact that the topic of egg freezing is currently receiving more attention on social media, including in Jordan (Sharaydih et al., 2025).

The study findings showed that a good fraction of women were positive about egg freezing (42.8%), while only 11.5% were opposed. These findings indicate that women in Jordan are open-minded to egg freezing. A previous Jordanian study reported a mean attitude score of 26.9 out of 44 among surveyed women, a result closely aligned with the findings of the current study (Sharaydih et al., 2025). In Jordan, assisted reproductive technologies, including gamete and embryo freezing, receive widespread social acceptance and are actively encouraged within the country's traditions as vital tools for supporting family formation (Albeitawi et al., 2026). This widespread cultural embrace is deeply tangled with Sharia law, which provides a strict yet supportive regulatory framework. Under these legal and religious guidelines, procedures are permissible exclusively within the bounds of a valid marriage using the couple’s own genetic material, ensuring that the lineage remains protected (Shestak et al., 2023). The proportion of positive attitudes reported in the current study is lower compared to studies from Iran (93.3%) and Ghana (68%) (Akhondi et al., 2023; Osaah et al., 2024), and is slightly higher than those reported in studies from Lebanon (34.1%), Germany (33%), the United Kingdom/Denmark (27%), the United States (21.6%), and Italy (19.5%) (Ghazeeri et al., 2023; Lallemant et al., 2016; Milman et al., 2017; Schick et al., 2017; Tozzo et al., 2019). These differences between different populations can be attributed to differences in culture, social norms, and sample characteristics.

The results showed a high level of consensus regarding the use of egg freezing to preserve reproductive choices under certain medical conditions, such as surgical manipulations of the ovaries, chronic illnesses, and cancer treatment. Overall, medical egg freezing saw higher acceptance rates than freezing for psychological or social reasons, such as work commitments and advanced maternal age. This agrees with a previous study from Jordan and investigations conducted in Australia, Sweden, Canada, and Lebanon (Daniluk & Koert, 2016; Ghazeeri et al., 2023; Johnston et al., 2020; Sharaydih et al., 2025; Wennberg et al., 2016). Regarding advanced maternal age, the current study showed that approximately half of the participants agreed that egg freezing is important for women who have reached advanced maternal age without having children (54.1%). This is consistent with the fact that the number of eggs declines sharply in women after the age of 35 (Jackson, 2016; Lewis et al., 2016). A similar pattern of agreement regarding social egg freezing was reported in a recent study from Lebanon, where 41.5% of participants agreed that egg freezing was used by single women who were unable to secure a right partner before reaching advanced maternal age (Ghazeeri et al., 2023). This consistency in findings across Middle Eastern populations suggests that the public is more likely to view partner-related childbearing as legitimate, compared to career-related decisions. Comparisons with developed countries such as Australia, the United Kingdom, and Denmark support this trend, showing greater support for egg freezing for partner-related reasons than for career advancement (Johnston et al., 2020; Lallemant et al., 2016). Interestingly, the United States is an exception, with a higher acceptance rate for career commitment than for delaying finding a suitable partner. This discrepancy may stem from societal differences in the importance of career advancement compared to motherhood (Mertes & Pennings, 2012).

Beyond assessing the acceptability of egg freezing, this study examined the concerns surrounding this technology. Key concerns included a lack of knowledge about the procedure, the risks of bleeding, infection, and hormones, the potential loss of virginity as a result of the procedure to remove eggs from the ovaries, and conflict with a future partner as key apprehensions associated with egg freezing. The majority of women expressed concerns about the safety of egg storage, fearing egg mixing and misuse, indicating that despite medical advances, many still view egg freezing as a risky or experimental procedure. Prior research in Jordan identified technology costs, future husband rejection, and social exclusion as primary concerns (Sharaydih et al., 2025). These concerns have also been reported in previous studies covering different populations (Ghazeeri et al., 2023). Such concerns offer a plausible explanation for the study's high rate of “maybe” responses regarding the technology.

The current study showed that age, higher education, higher income, marital status, and having children were factors associated with acceptance of egg freezing. Women typically bear the costs of egg freezing for fertility preservation in non-medical situations. However, the costs of egg freezing are an important factor for an individual who may decide to use this technology (Mertes & Pennings, 2012; Sharaydih et al., 2025). This is consistent with the current finding, where acceptance of egg freezing was associated with higher income, and contradicts a previous study in Lebanon, where financial factors were the least important factor (Ghazeeri et al., 2023). In a study of Danish and British women's attitudes, cost was not a determining factor in women's decisions, with the majority of responses being positive whether or not the procedure was funded (Lallemant et al., 2016). The current study also showed that education was a significant factor in the decision to freeze eggs. Participants with higher education showed higher acceptance rates than university students, which is consistent with what was observed in the Lebanese study, where a high acceptance rate was associated with a higher proportion of participants with advanced degrees (Ghazeeri et al., 2023). However, in a Danish and British study, education level was not significantly associated with egg freezing (Lallemant et al., 2016). Among the other examined variables, marital status and having children were found to be associated with acceptance of this technology. This contrasts with previous literature that has shown that single women consistently showed greater support or willingness to consider egg freezing and that women without children were more likely to express interest in elective egg freezing than women who already had children (Alteri et al., 2019; Chehimi et al., 2025; Ghazeeri et al., 2023; Gullo et al., 2022; Johnston et al., 2020). The discrepancy between previous literature and the current Jordanian study can be attributed to the nature of the Jordanian population, where family size is large, and women desire to have a number of children (Gharaybeh, 2014).

Among the study implications, for healthcare providers, findings highlight the need for targeted educational counseling that addresses specific concerns regarding procedural risks (e.g. bleeding, infection, and hormonal impact) and storage safety, while proactively discussing age-related fertility decline and elective egg freezing with younger and less-educated women. For policymakers, the high acceptance of medical egg freezing underscores the urgency of establishing clear regulatory frameworks to prevent sample misuse and expanding public health insurance or institutional coverage for women facing reproductive threats such as cancer, chronic illnesses, or premature menopause.

Limitations of this study include its reliance on non-probability convenience and snowball sampling methods via social media, which introduces substantial selection and coverage biases. This approach likely skews the sample toward younger, more educated, urban, and digitally active individuals, systematically excluding Jordanian women without social media access or specific group memberships. Furthermore, because a snowballing chain network lacks a defined sampling frame, the exact response rate could not be calculated, limiting the generalizability of the findings to the wider population of Jordanian women. Additionally, the study relies on self-reported data subject to potential response bias, lacks qualitative anecdotal data to explain unexplored situations, and utilizes a cross-sectional design that precludes establishing causal associations. To better capture Jordanian women's opinions on this technology, future research should utilize probability-based sampling methods, such as stratified random sampling, to recruit a more representative and diverse national sample of Jordanian women that includes rural and digitally disconnected populations.

## Conclusion

Jordanian women view egg freezing positively, showing greater acceptance for medical than social reasons. Future initiatives should integrate fertility education into public health, improve technology access, and support evidence-based decisions that respect personal autonomy.

## Data Availability

Raw data are available upon request from the corresponding author.
